# Topoisomerase Inhibitors Modulate Gene Expression of B-Cell Translocation Gene 2 and Prostate Specific Antigen in Prostate Carcinoma Cells

**DOI:** 10.1371/journal.pone.0089117

**Published:** 2014-02-20

**Authors:** Kun-Chun Chiang, Ke-Hung Tsui, Li-Chuan Chung, Chun-Nan Yeh, Phei-Lang Chang, Wen-Tsung Chen, Horng-Heng Juang

**Affiliations:** 1 Department of General Surgery, Chang Gung Memorial Hospital, Keelung, Taiwan, ROC; 2 Department of Urology, Chang Gung Memorial Hospital, Kwei-Shan, Tao-Yuan, Taiwan, ROC; 3 Department of Anatomy, College of Medicine, Chang Gung University, Kwei-Shan, Tao-Yuan, Taiwan, ROC; 4 Department of General Surgery, Chang Gung Memorial Hospital, Kwei-Shan, Tao-Yuan, Taiwan, ROC; 5 National Kaohsiung University of Hospitality and Tourism, Hsiao-Kang, Kaohsiung Taiwan, ROC; University of Kentucky College of Medicine, United States of America

## Abstract

Camptothecin (CPT) and doxorubicin (DOX) have been demonstrated to have potent anti-tumor activity. The B-cell translocation gene 2 (BTG2) is involved in the regulation of cell cycle progression. We evaluated the molecular mechanisms of CPT and DOX on cell proliferation and the expressions of BTG2 and prostate specific antigen (PSA) in prostate carcinoma cells. Our results indicated that CPT or DOX treatments induced Go/G1 cell cycle arrest in LNCaP cells and apoptosis at higher dosage. Immunoblot and transient gene expression assay indicated that CPT or DOX treatments induced p53 and BTG2 gene expression, with the later effect dependent on the p53 response element within BTG2 promoter area since mutation of the p53 response element from GGGAAAGTCC to GGAGTCC or from GGCAGAGCCC to GGCACC by site-directed mutagenesis abolished the stimulation of CPT or DOX on the BTG2 promoter activity, which is also supported by our results that cotreatments of pifithrin-α, an inhibitor of p53 dependent transcriptional activation, blocked the induction of CPT or DOX on BTG2 gene expression. CPT or DOX also downregulated the protein expressions of androgen receptor (AR) and PSA. Transient gene expression assays suggested that CPT or DOX’s attenuation of PSA promoter activity is dependent on both the androgen and p53 response elements within of the PSA promoter. Our results indicated that CPT and DOX attenuate cell proliferation via upregulation of BTG2 gene expression through the p53-dependent pathway. The CPT and DOX block the PSA gene expression by upregulation of p53 activity and downregulation of androgen receptor activity.

## Introduction

Prostate cancer, the second most common solid tumor for men in United States, has approximately 41,740 new cases diagnosed and 28,170 dying of this disease in 2012 [1]. The recent improvement of prostate-specific antigen (PSA) measurement leads to the early diagnosis of prostate cancer more possibly and, thus, improves the survival while increasing the incidence. However, for the high-risk prostate cancer patients, accounting for 15% of all prostate cancer, 30–60% of them would develop biochemical recurrence (BCR), flare up of PSA, at around 10 years [2], followed by the clinical metastasis months to years later [3]. Even under the state-of-art management for high risk prostate cancer patients, which include multimodal approaches, 10–25% prostate cancer patients die of metastatic disease [3]. Thus, to develop a new therapeutic regimen against prostate cancer is urgently needed.

Topoisomerases, a family of highly conserved enzymes, play an important role during DNA topology and exist ubiquitously in all eukaryotic cells. Camptothecin (CPT) and its derivatives, belonging to one kind of selective topoisomerase I (TOP1) inhibitor, repress cell proliferation through fixing TOP1 by forming an irreversible TOP1cleavable complex, and,thus, have been applied for anticancer treatment for about 15 years [4]. Doxorubicin (DOX), the commonly used chemotherapeutic drug, exerts its antitumor effect through inhibiting TOP I and II to intervene in DNA uncoiling [5].

The B-cell translocation gene 2 (BTG2), belonging to antiproliferative APRO family proteins [6], preferentially expresses in quiescent cells and its antiproliferative effect is partly mediated by p53 dependent component of the DNA damage cellular response pathway. Our previous in vitro study revealed that expression of BTG2 is related to neoplasia of human prostate carcinoma cells and forced-overexpression of BTG2 in prostate carcinoma PC-3 cells attenuated cell proliferation [7].

The prostate specific antigen (PSA), a 30- to 33 kDa glycoprotein, is expressed in all stages of prostate cancer and primarily regulated by androgen. The distinct characteristic of PSA that it is produced almost exclusively by the luminal epithelial cells of the human prostate makes it a well-known biomarker for diagnosing and evaluating the status of prostate cancer [8]. Previously, PSA has been identified as the p53-downstream gene [9].

Studies have indicated that LNCaP cells are CPT-sensitive prostate carcinoma cells, and that CPT-induced apoptosis is associated with an increase in p53 expression in intestinal epithelial cells [10], [11]. Our objectives for this study were to determine the effects and regulatory mechanisms of CPT and DOX on the gene expression of BTG2 and PSA in prostate cancer cells.

## Materials and Methods

### Materials, Cell lines, and Cell Culture

LNCaP and PC-3 cell lines were obtained and maintained as described previously [12]. CPT, DOX, pifithrin-α, and MG132 were purchased from Sigma (St. Louis, MO). The RPMI-1640 culture media were purchased from Life Technologies (Rockville, MD). Phenol red free RPMI 1640 (RPMI-PRF) media were purchased from AppliChem (Darmstadt, Germany), and fetal calf serum (FCS) was purchased from the HyClone (Logan, Utah). LNCaP and PC-3 cells were cultured in RPMI 1640 medium with 10% FCS. The CD-FCS was considered as charcoal-dextran-treated FCS by using the charcoal to remove the steroids.

### Cell Proliferation Assay

Cell proliferation in response to (S)-(+)-CPT and DOX was measured using a ^3^H-thymidine incorporation assay as previously described [13].

### Assessment of Apoptosis by Flow Cytometry

Cells were serum starved for 24 hours and then cultured in RPMI 1640 medium with 10% FCS and with or without different concentrations of drugs for another 24 hours. The cells were then collected and stained with propidium iodide. Cell cycle analysis was performed using the FACS-Calibur cytometer and CellQuestPro software (BD Biosciences, San Jose, CA); the data were analyzed using ModFit LT Mac 3.0 software as previously described [14].

### Immunoblot Assay

LNCaP cells were incubated in the RPMI-PRF medium with 10% FCS and different drugs for a period of 24 hours. Equal quantities of cell extract (20–40 µg) were loaded onto a 12% sodium dodecyl sulfate polyacrylamide (SDS) gel and analyzed by the electrochemiluminescent detection system. Polyclonal rabbit anti-human BTG2 serum was prepared as previously described [7]. The blotting membranes were probed with 1∶500 diluted polyclonal PSA antiserum (A0562, DakoCytomation, Glostrup, Denmark), 1∶200 diluted human androgen receptor antiserum (N-20; Santa Cruz Biotechnology, Santa Cruz, CA), 1∶500 diluted human p53 antiserum (Santa Cruz Biotechnology), or 1∶3000 diluted β-actin antiserum (I-19, Santa Cruz Biotechnology). The intensity of different bands were analyzed by GeneTools of ChemiGenius (Syngene, Cambridge, UK).

### Expression Vector and Report Vector Constructs

**Figure 1:**
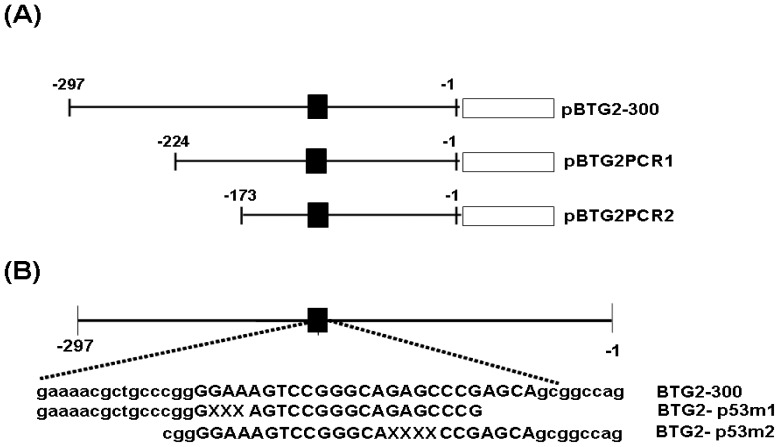
Map of BTG2 reporter vectors. (A) Nested deletion constructs of pBTG2-300, pBTG2-PCR1 and pBTG2-PCR2. ▪ represents the region of p53 response element. (B) The two p53 response element mutants of BTG2 reporter vectors (pBTG2-p53m1 and pBTG2-p53m2) were constructed by site-directed mutagenesis. Uppercase indicates consensus sequence of p53 response element and “X” represents the deletion sites.

The p53 expression vector and MMTV reporter vector were constructed as previously described [15], [16]. The 300 bp 5′-flanking region (−1 to −297) of the human BTG2 gene was cloned as previously described [7]. The reporter vectors containing the 5′-deletion fragment and mutant p53 response element of BTG2 gene were established using a PCR and QuikChange site directed mutagenesis kit (stratagene, La Jolla, CA). The constructing map is showed in the Figure 1. The enhancer/promoter of the PSA gene was isolated from the PAC clone (LLNL-214C7; Human Genome Mapping Project Resource Centre, UK). The reporter vectors (pPSAH: −41 to −5874; pPSABHE, −4801 to −3933 and −41 to −589; pPSABHp53m and pPSABHEp53m) containing 5′-flanking region of the human PSA gene were cloned by 5′-deletion or site direct mutagenesis as previously described [9], [17].

### Luciferase and β-Galactosidasea Assay

Cells were seeded onto 24-well plates at 1×10^4^ cells/well 1 day prior to transfection. The cells were transiently transfected using TransFast transfection reagent (0.6 µg/well; Promega Bioscience, San Luis Obispo, CA) with 1 µg/well of reporter vectors as indicated and 0.5 µg/well of β-galactosidase expression vector (pCMVSPORTβgal; Life Technologies, Rockville, MD) in the OPTI-medium (Life Technologies) as described previously [18]. After 6–8 hours, the media containing the liposome-DNA complex was removed and replaced with RPMI 1640 medium with 5%CD-FCS for overnight. The media were replaced with the same media as described above but contained different concentrations of drugs. After further 24 hours incubation, cells were harvested and activities of luciferase and β-galactosidase were assayed as specified by the manufacturer instructions (Promega Bioscience).

### Statistical Analysis

Results are expressed as the mean ± S.E. of at least three independent replication of each experiment. Statistical significance was determined by one-way ANOVA and Student’s pair-*t* test using the SigmaStat program for Window version 2.03 (SPSS Inc, Chicago, IL).

## Results

**Figure 2:**
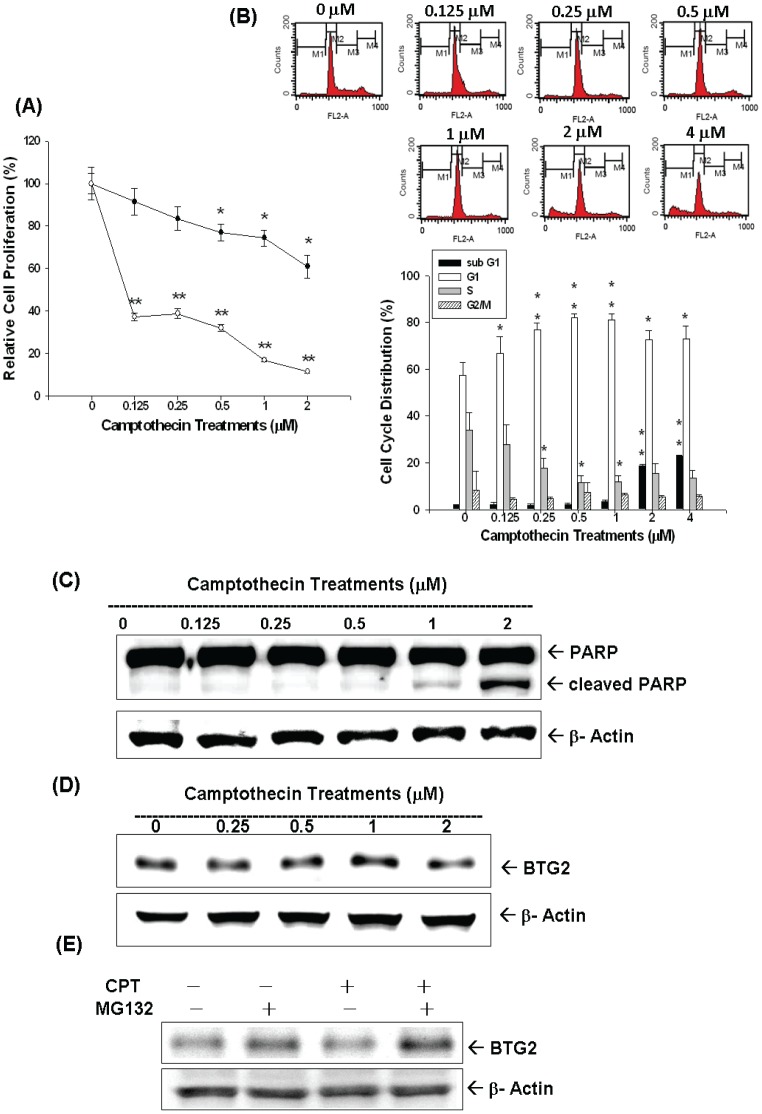
Camptothecin (CPT) regulates cell proliferation and cell cycle by modulating BTG2 expression in LNCaP cells. (A) LNCaP cells were treated with various concentrations of CPT treatments for 24 (black circle) and 48 (white circle) hours and the cell proliferation was determined by the ^3^H-thymidine incorporation. (B) LNCaP cells were serum starved for 24 hours and then were treated with 0–4 µM of CPT as indicated for 24 hours. The cells were stained with PI, and the cell cycle distribution was analyzed by flow cytometry. Each box represents the mean+SE (n = 6). LNCaP cells were treated with various camptothecin treatments as indicated for 24 hours. Cells were lysed and expression of PARP, cleaved PARP (c-PARP) (C) and BTG2 (D) were determined by immunoblot assay. (E) LNCaP cells were pretreated with MG132 and then treated with 2 µM CPT for 24 hours. Cells were lysed and expression of BTG2 was determined by immunoblot assay.

Cell proliferation of LNCaP cells in response to different concentrations of CPT treatment for 24 and 48 hours, respectively, was measured by ^3^H-thymidine incorporation assay. Results indicated cell proliferation decreased 30% when cells were treated with 2 µM of CPT for 24 hours; however, cell proliferation decreased more than 60% after treatment with 0.125–2 µM of CPT for 48 hours (Figure 2A). Results from flow cytometric analysis of treated LNCaP cells revealed that low dose CPT (0–1 µM) induced cell cycle arrest at G0/G1 dose-dependently. 1 µM CPT induced∼20% increase in G0/G1 phase cell together with a decrease in S phase cells after 24 hours incubation in LNCaP cells (Figure 2B). High dose of CPT (2 to 4 µM) induced cell apoptosis indicated by the 15–20% increase of sub-G1 fraction of cells (Figure 2B), which was supported by immunoblot assay result which revealed that treatment with 1–2 µM of CPT induced the expression of cleaved form of PARP in LNCaP cells (Figure 2C). The expression of BTG2 in LNCaP cells increased after 0–1 µM CPT treatments; however, the protein levels of BTG2 decreased by 2 µM CPT treatment (Figure 2D). Since high dose of CPT (2–4 µM ) induced apoptosis (Figure 2B), which, in turn, may cause the degradation of BTG2 protein via ubiquitin-proteason system, we then cotreated the MG132, a proteasome inhibitor, and CPT in LNCaP cells. As shown in Figure 2E, MG132 partially restored the 2 µM CPT-induced BTG2 protein expression.

**Figure 3:**
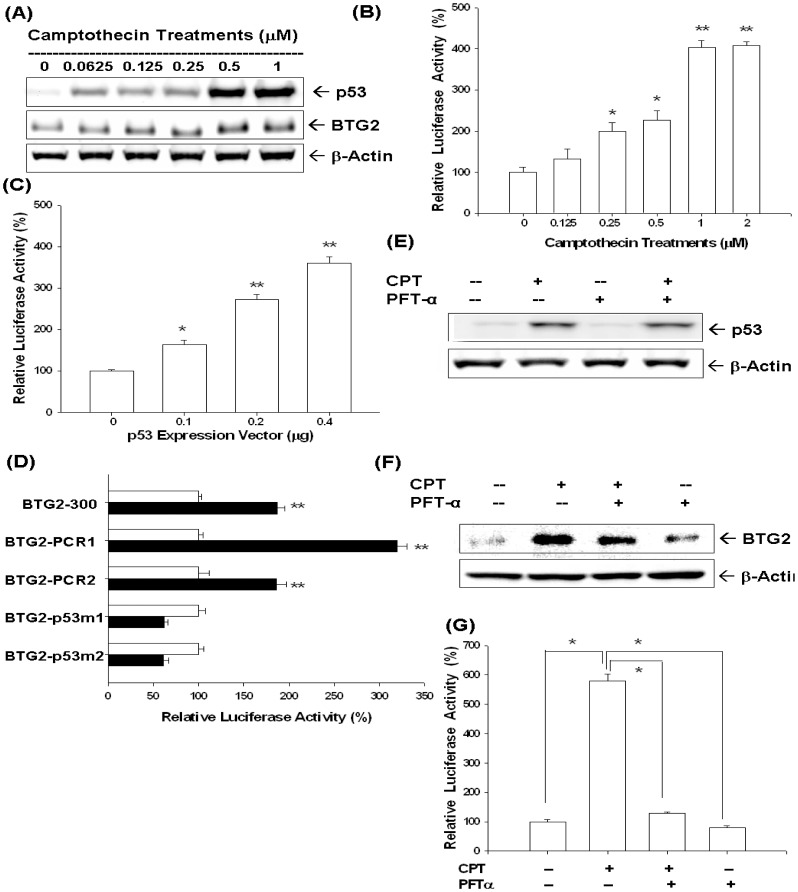
Camptothecin (CPT) upregulates BTG2 expression dependent on p53 response element of the human BTG2 gene in LNCaP cells. (A) LNCaP cells were treated with indicated concentrations of CPT for 24 hours. Cells were lysed and expressions of BTG2 and p53 were determined by immunoblot assay. (B) Luciferase activity of BTG2 reporter vector (pBTG2-300)-transfected LNCaP cells treated with different concentrations of CPT. (C) Luciferase activity of BTG2 reporter vectors (pBTG2-300) with different concentrations of p53 expression vector-contrasfected LNCaP cells. (D) Luciferase activity of nested deletion or mutation constructs of BTG2 reporter vectors-transfected LNCaP cells after treatment with control solvent (white bars) or 1 µM of CPT (black bars). Data are presented as the mean percentage+SE (n = 6) of the BTG2 reporter activity induce by CPT treatment in relation to the control solvent-treated group (*P<0.05, **P<0.01). LNCaP cells were cotreated with 1 µM CPT and 30 µM pifithrin-α (PFT-α). Cells were lysed and expression of p53 (E) and BTG2 (F) were determined by immunoblotting assay. (G) Luciferase activity of BTG2 promoter vector-transfected LNCaP cells treated with 1 µM of CPT or/and 30 µM PFT-α (*P<0.01).

As shown in Figure 3A, in addition to BTG2, CPT, from 0 to 1 µM, also increased LNCaP cells p53 expression in a dose dependent manner. Moreover, the promoter activities of BTG2 gene were upregulated by CPT treatments in LNCaP cells (Figure 3B) or by transient overexprssion p53 in p53-null PC-3 cells (Figure 3C). Transient gene expression assay with site-directed mutagenesis indicated that CPT affects BTG2 gene expression via the p53 response elements located at human BTG2 promoter (Figure 3D). Western blot results revealed pifithrin-α treatment (30 µM), an inhibitor of p53 dependent transcriptional activation, did not affect the expression p53 but blocked the induction of CPT on BTG2 expression (Figure 3E, F). The transient gene expression assays showed similar results indicating the pifithrin-α blocked the activation of CPT on BTG2 promoter activity (Figure 3G), in line with the result shown in Figure 3D.

**Figure 4:**
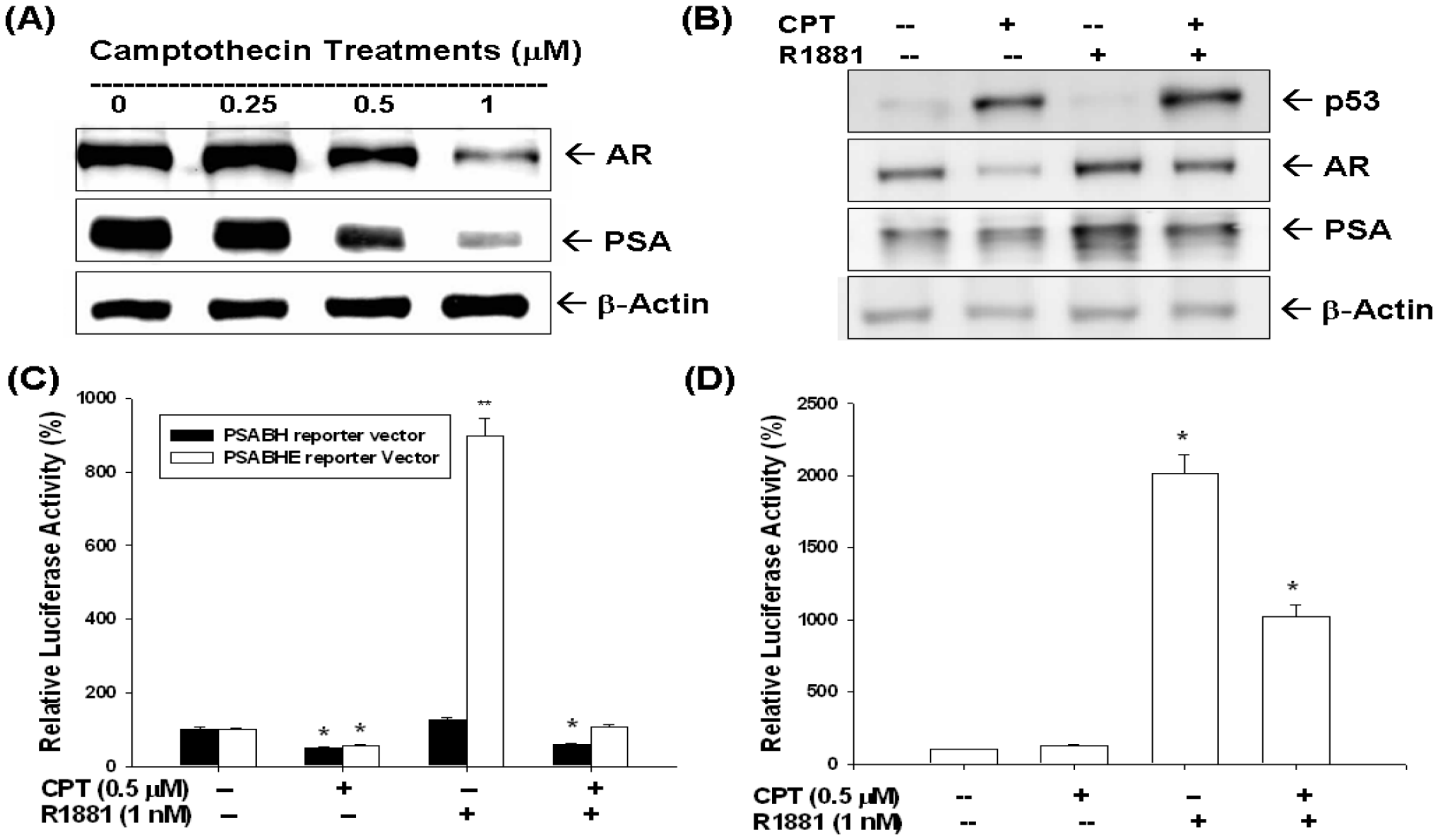
Camptothecin (CPT) modulates p53, androgen receptor and prostate specific antigen expression of LNCaP cells. LNCaP cells were treated with different concentrations of CPT for 24 hours. Cells were lysed and androgen receptor (AR) and prostate specific antigen (PSA) expressions were determined by immunoblotting assays. (B) LNCaP cells were treated with 1 µM CPT or/and 1 nM R1881. Cells were lysed and expression of p53, AR, and PSA were determined by immunoblotting assay. (C) The pPSABH-transfected LNCaP cells (black bars) or the pPSABHE-transfected LNCaP cells (white bars) were treated with 0.5 µM CPT or/and 1 nM R1881 for 24 hours. Data are expressed as the mean percentage±SE of the stimulations of the PSA reporter activity. (D) Luciferase activity of MMTV reporter vector-transfected LNCaP cells after treatment with 0.5 µM CPT or/and 1 nM R1881 for 24 hours. Data are expressed as the mean percentage±SE of the stimulations of the MMTV reporter activity (*P<0.01).

Moreover, CPT also blocked the AR and PSA expression determined by immunoblotting assay (Figure 4A). When LNCaP cells were treated with or without 1 nM R1881and CPT (0.5 µM) in RPMI-PRF medium with 5% CD-FCS for 24 h, the results of immunoblot assay revealed that CPT blocked the stimulation of R1881 (1 nM) on AR and PSA expression (Figure 4B). Transient gene expression assays indicated that R1881 (1 nM) induced PSA promoter activity when using the reporter vector (pPSABHE) containing the promoter (−41 to −589) and enhancer (−4801 to −3933), which included the androgen response element (ARE) DNA fragment, but not the reporter vector (PSABH) containing only the promoter (−41 to −589) DNA fragment (Figure 4C). The CPT not only blocked the reporter activity of PSA gene but also attenuated the stimulation of R1881 (1 nM) on PSA gene expression (Figure 4C). We also confirmed that CPT blocked the AR activity by using the reporter vector that contained androgen-sensitive MMTV promoter fused to a luciferase reporter gene (Figure 4D).

**Figure 5:**
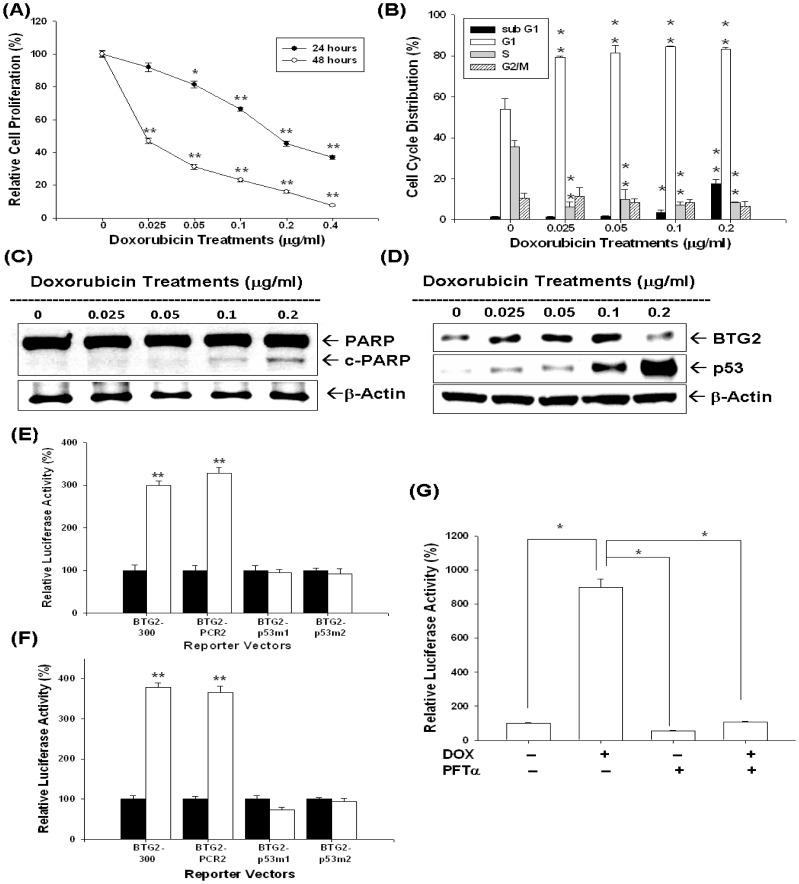
Doxorubicin (DOX) modulates cell proliferation and cell cycle progression through upregulation of BTG2 p53 dependently in LNCaP cells. (A) LNCaP cells were treated with indicated concentrations of DOX for 24 (black circle) and 48 (white circle) hours, respectively, and the cell proliferation was determined by the H3-thymidine incorporation. (B) LNCaP cells were serum starved for 24 hours and then treated with 0–0.2 µg/ml of DOX for 24 hours. The cells were stained with PI, and the cell cycle distribution was analyzed by flow cytometry. Each box represents the mean±SE (n = 6). LNCaP cells were treated with various concentrations of DOX as indicated for 24 hours. Cells were lysed and expression of PARP, cleaved PARP (c-PARP) (C), p53 and BTG2 (D) were determined by immunoblotting assays. (E) Luciferase activity of different kinds BTG2 reporter vectors-transfected LNCaP cells after treatment of 0.1 µg/ml of DOX (white bars) or control-solvent (black bars). (F) Luciferase activity of different kinds of BTG2 reporter vectors with p53 expression vector (white) or pcDNA3 control vector (black bars)-contransfected LNCaP cells. (G) Luciferase activity of BTG2 promoter vector (pBTG2-300)-transfected LNCaP cells treated with DOX or/and 30 µM pifithrin-α (PFT-α) (*P<0.01). Data are presented as the mean percentage±SE (n = 6) of the BTG2 reporter activity induce by drug treatments in relation to the control solvent-treated group (*P<0.05, **P<0.01).

Results from ^3^H-thymidine incorporation assay indicated cell proliferation decreased 55% when cells were treated with 0.2 µg/ml of DOX for 24 hours; however, cell proliferation decreased more than 53% after treatment with 0.0125–0.4 µg/ml of DOX for 48 hours (Figure 5A). Results from flow cytometric analysis of LNCaP cells revealed that 0.05–0.2 µg/ml of DOX induced around 20% increase in G0/G1 phase cells together with a decrease in S phase cells after 24 hours incubation. DOX (0.2 µg/ml) also increased the sub-G1 fraction of cells by 17% (Figure 5B). Immunoblot assay indicated that treatment with 0.1–0.2 µg/ml of DOX induced the expression of cleaved form of PARP in LNCaP cells (Figure 5C). Immunblot assay also revealed that expression of BTG2 and p53 in LNCaP cells increased after DOX, from 0.025 to 0.1µg/ml, treatments (Figure 5D). Transient gene expression with site-directed mutagenesis using DOX-treated LNCaP cells (Figure 5E) or transient p53-overexpressed PC-3 cells (Figure 5F) indicated that DOX affects BTG2 gene expression via the p53 response elements within BTG2 promoter, also supported by the result that pifithrin-α treatment (30 µM) blocked the induction of DOX on BTG2 promoter activity (Figure 5G).

**Figure 6:**
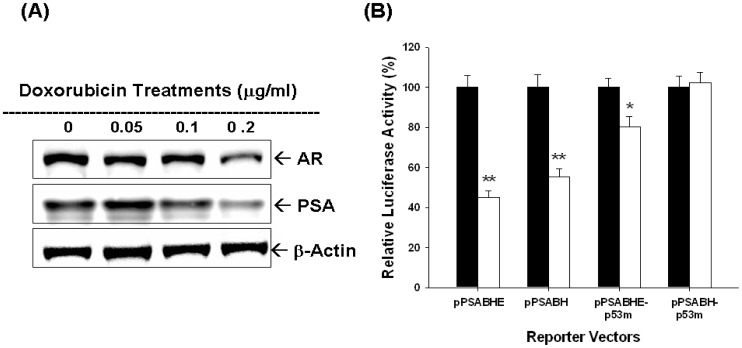
Doxorubicin (DOX) modulates PSA expression dependent on the activities of androgen receptor and p53 in LNCaP cells. (A) Cells were treated with indicated concentrations DOX for 24 hours. Cells were lysed and expression of androgen receptor (AR) and PSA was determined by immunoblotting assays. (B) Luciferase activity of different kinds of PSA reporter vectors transfected-LNCaP cells treated with control solvent (blacke bars) or 0.1 µg/ml of DOX (white bars). Data are presented as the mean percentage±SE (n = 6) of the PSA reporter activity induced by DOX in relation to the control solvent-treated group (*P<0.05, **P<0.01).

Results of immunblotting assays also indicated that DOX treatments blocked the expressions of AR and PSA (Figure 6A). To investigate how DOX affected PSA gene expression, we next applied 4 different PSA reporter vectors, including pPSABHE, pPSABH, pPSABHE-p53m, and pPSABH-p53m. The transient gene expression assays confirmed that downregulation of DOX on PSA promoter activity may not only dependent on p53 response element on the PSA promoter region (−41 to −589) but also the androgen response element of enhancer region (−4801 to −3933) (Figure 6B).

## Discussion

CPT and DOX are widely used as anticancer regimens clinically. Both drugs belong to the TOP inhibitors and disturb DNA uncoiling leading to arrest of cell duplication [4], [5]. In this study, as shown in figure 2A, 2B, 5A, and 5B, we clearly demonstrated that both CPT and DOX repressed LNCaP cell growth through induction cell cycle arrest at G0/G1 (lower concentration), indicated by increased G0/G1 cell population, and apoptosis (higher concentration ) as indicated by increased sub-G1 cell population and cleaved PARP (Figure 2C and 4C).

BTG2, belonging to antiproliferative (APRO) genes together with BTG1, BTG3/ANA/Rbtg3 and Tob genes, is first known as one kind of the early growth response genes [19]. It was later isolated from 3T3 fibroblasts cells [20]. BTG2 plays a vital role in regulation of cell cycle transition. It can disturb cell cycle process at G1/S or G2/M phases in cancer cells with inactive pRB and/or p53 and function as a pan-cell cycle modulator in a cell- or tissue- specific manner [21]. The induction of BTG2 comprises at least two pathways including wild type p53 [22], [23], or p53 independent PKC-δ pathway [24]. Previous studies have shown that BTG2 did play an important role to repress cell growth in gastric, breast, bladder, and prostate carcinoma cells [7], [14], [18], [25]–[27].

p53 is a well-known tumor suppressor gene and mutations in the p53 gene have been identified in a variety of human cancers including prostate cancer [28]. Our previous studies indicated that p53 is the target gene of CPT and DOX in LNCaP cells [9], [15]. In this current study, we found that both CPT and DOX could induce p53 and BTG2 expression in LNCaP cells (Figure 2D, 3A and 5D). To evaluate whether the induction of BTG2 by CPT and DOX is p53-dependent or not, we first measured the promoter activities of BTG2 gene after CPT or DOX treatment in p53-wild type LNCaP cells or transient overexprssion p53 in p53-null PC-3 cells. Our results clearly showed that both CPT or DOX treatment or transient overexpression p53 could upregulate BTG2 promoter activity (Figure 3C, 3D, 5E and 5F). Since our previous report and study from other independent laboratory using simple DNA analysis indicated that BTG2 promoter area contains a putative p53 response element (RRRCWWGYYYN_(0–13)_RRRCWWGYYY) [7], [29], we next conducted site-directed mutagenesis with transient BTG2 reporter vector expression assay. As shown in Figure 3D and Figure 5E, both BTG2-p53m1 and BTG2-p53m2 had the reduced CPT- or DOX-induced luciferase activity as compared to wild-type BTG2 reporter vectors, which suggested CPT and DOX induced BTG2 expression through p53 signal pathway. To further verify this, we applied pifithrin-α, an inhibitor of p53 dependent transcriptional activation, and found pifithrin-α blocked BTG2 expression but not p53 expression in LNCaP cells after CPT treatment. The transient gene expression assay indicated that pifithrin-α blocked CPT or DOX induced BTG2 expression in LNCaP cells (Figure 3 and Figure 5). Taken together, we thus concluded that both CPT and DOX induced BTG2 expression in LNCaP cells through the p53-dependent pathway. Of note, as shown in figure 2D, BTG2 protein level was repressed instead of upregulated by 2 µM CPT. Our explanation is since 2–4 µM CPT induced apoptosis in LNCaP cells, which may lead to BTG2 degradation via ubiquitin-proteason system, the CPT-induced BTG2 protein was degraded. To confirm this, we cotreated CPT with MG132, a proteasome inhibitor, together in LNCaP cells and found the CPT-induced BTG2 protein expression was restored (Figure 2F). These results are consistent with early report which indicated that proteins of APRO family are sensitive to the ubiquitin-proteasome system [30].

PSA, a 33-kDa glycoprotein, is present in normal hypertrophic and malignant prostatic tissue, and is later to be found detectable in human serum and elevated in patients with prostate cancer [31]. After treatment, PSA was proved to be a sensitive biomarker to detect prostate cancer recurrence [8], [32]. Studies have reported that PSA may facilitate refractory prostate tumor progression and may be involved in the invasion of prostate cancer as well [33], [34]. To date, the utilization of serum PSA has indeed revolutionized current management and detection of prostate cancer. PSA has been deemed as an independent prognostic marker for prostate cancer patients during long-term follow up because most patients have increased PSA levels years before prostate cancer is diagnosed clinically. *In vitro* studies have demonstrated that several transcriptional factors, like androgen receptor (AR), c-Myc-associated zinc finger protein (MAZ), activator of the transcription 3 (STAT3), prostate-derived Ets factor (PDEF), p53, NF-kB, and Sp family genes are found to involve the gene regulation of PSA, with most of the transcriptional factor interacting with androgen receptor to modulate PSA promoter activity [9], [17], [18], [35]–[37].

In this study, we showed that CPT and DOX modulated not only the p53, AR and PSA protein expression but also R1881-induced PSA expression in LNCaP cells (Figure 4A and 4B). In addition, the AR activity was also blocked by CPT as shown by using the reporter vector with androgen-sensitive MMTV promoter (Figure 4D). Since PSA promoter area has been shown to have p53 and AR response elements [9], [17], we next conducted transient gene expression assays and found that downregulation of PSA by DOX was abolished as we took ARE away and mutated p53 response element in the PSA reporter vector (Figure 6B). We thus concluded that downregulation of doxorubicin on PSA promoter activity may not only dependent on p53 response element on the PSA promoter region (−41 to −589) but also the androgen response element of enhancer region (−4801 to −3933) (Figure 6B). These results provide the first evidence to demonstrate that TOP inhibitors modulate the PSA gene expression via both p53 and AR activity in LNCaP cells.

In conclusion, TOP inhibitor, CPT and DOX, could inhibit prostate carcinoma LNCaP cells growth through cell cycle arrest at G0/G1 and apoptosis induction. TOP inhibitors induce BTG2 expression in a p53-dependent pathway and repressed PSA expression both p53 and AR dependently.
